# Abstract of Paper Read by Reginald H. Sayre, M, D., before the Pan American Medical Congress, Washington, Sept. 1893

**Published:** 1893-10

**Authors:** 


THE
### Southern Medical Record.

A MONTHLY JOURNAL OF MEDICINE AND SURGERY.

VOL. XXIII. ATLANTA, GA., OCTOBER, 1893. No. 10

### Original Articles.

### ABSTRACT OF PAPER READ BY REGINALD H. SAYRE, M. D., BEFORE THE PAN-AMERICAN MEDICAL CONGRESS, WASHINGTON, SEPTEMBER, 1893.

#### A CONTRIBUTION TO THE STUDY OF CLUB-HAND.

Club-hand is very much less frequent than club-foot. It may be acquired as the result of paralysis of certain muscles or contraction of others from central nervous irritation, by cicatrices resulting from burns, or be due to injuries to the bones of the hand or forearm, or it may be congenital.

Of the first variety, J. K. Young reports a case where an infant had the left side of the head injured at birth. A large hematoma formed here, and subsequently the right hand was markedly adducted and the fingers and thumb flexed, and the hand flexed at the wrist, almost at a right-angle with the forearm in the radio-palmar position. The hematoma was incised, profuse bleeding followed, and subsequently the deformity gradually subsided, having been caused by the irritation produced by the hematoma.

Biehaut reports a case of club-hand due to fracture of the ulnar at birth, with subsequent loss of bone from suppuration, giving rise to inequality in the length of the bones of the forearm, causing a sharp deflection of the hand towards the ulnar side.

The congenital club-hands differ widely from the above described cases, and may be divided into three varieties: first, those where the skeleton is complete and well-formed; second, where the skeleton is complete but ill-formed; and third, where the skeleton is incomplete and distorted. Various writers say that the majority of cases come under the third head, but the author's personal experience does not agree with this.

In many cases club-hand is associated with club-foot or some other abnormality of development. The direction of the deformity may be either in flection, extension, abduction or adduction, or a combination of the two, the most frequent being the radio-palmar variety.

In those cases where all the bones of the hand and forearm are present, the prospects of a good result are more favorable than where there is absence of one or more bones, and in these milder cases, when seen early, it is sometimes possible to restore the hand to proper shape and function by constant manipulation and rotation of the parts, which are to be held in their improved position by some fixed dressing, as the Plaster-of-Paris bandage, the dressing being changed from time to time as the deformity is reduced.

Section of the tendons, ligaments or fascia may be necessary if the case is not seen in the early stages. Many of these structures are so situated as to make open section preferable to the subcutaneous method, and if the flexor tendons have to be divided, it would seem better to operate in the forearm instead of the hand, and to split the tendons longitudinally, and after having gained such additional length as was needed by sliding the ends past each other, to suture them together once more.

In an aggravated case of congenital club-hand and club-foot of the right side, associated with lateral curvature of the spine, the author had operated in the following manner: The club-hand was very marked. The radius and thumb were absent, as well as the first metacarpal bone and a certain number of the carpal bones. The ulnar was curved in its middle at an angle of about 30 degrees towards the side where the radius should have been. The hand was almost at right-angles with the forearm, bent towards the radial side, and flexed on the forearm. The carpus did not articulate with the ulnar, but was attached to it by means of firm ligamentous bands. An osteotomy was first done on the ulnar to correct the curve, and after the bone had united in a straight line, endeavors were made to stretch the contracted soft parts on the side of the arm where the radius should have existed. After several weeks of traction the hand could not be drawn far enough down to permit the ulnar to slide above the carpus. Through an open incision the ligaments between the ulnar and the carpus were divided, the intention being to form an artificial joint between the lower end of the ulnar and carpus. It was found impossible, however, to draw the carpus clear of the ulnar, and therefore the styloid process of the ulnar was cut off, the os magnum and unciform removed, and the end of the ulnar put into the gap in the carpus thus formed. The bones were not wired in this position, with the idea that the hand might be more useful if this were not done, and it being of course feasible to wire the bones later on, if it should be deemed necessary. The shortening of the extremity, caused by the removal of this amount of bone, seemed preferable to the author, to the very extensive division of tendons and muscles which would have been necessary to permit the carpus to be pulled down. The hand is now approximately in line with the forearm. There is free motion at the wrist, and the ability to grasp objects is greater than it was before the operation, although extension of the hand on the wrist is poor, absence of the radius making a very imperfect joint.

In cases like that described by Bouvier, which is in the Dupuytren Museum, where such carpus as is present articulates with the ulnar on the side where the radius should have been, the radius being absent, the proper operation would seem to be the division of the ulnar just above the articulation with the carpus, and then to turn it at right-angles letting the outer surface re-unite with the end of the ulnar, and thus bring the hand into a straight line with the arm, at the same time preserving the wrist-joint.

